# 

**Published:** 1897-01

**Authors:** 


### CONTENTS—JANUARY, 1897.—VOL. XII., NO. 7.

ORIGINAL CONTRIBUTIONS—

Medical Expert Testimony. B. M. Worsham, M. D . . . . . . . . . . . . . . . . . 351
The Use of Steam in Diseases of the Uterus. H. Leonards, M. D . . . . . . . . . . . . 356
Our Duty as Physicians. Robert E. Moss, M. D. . . . . . . . . . . . . . . . . . . . . . . 360
Dr. Osborn's Carbuncle Treatment. E. A. Morris, M. D . . . . . . . . . . . . . . . . . . 364

SOCIETY NOTES—

Richmond Academy of Medicine and Surgery . . . . . . . . . . . . . . . . . . . . . . 366
Central Texas Medical Association. . . . . . . . . . . . . . . . . . . . . . . . . . . . 374

ABSTRACTS AND SELECTIONS—

Suicide Considered a Mental Epidemic . . . . . . . . . . . . . . . . . . . . . . . . 376
Things Every Doctor Should Know . . . . . . . . . . . . . . . . . . . . . . . . . . 382
The "Mirror" Reflects . . . . . . . . . . . . . . . . . . . . . . . . . . . . . . . . . 384
Uterine Hemorrhage Following Abortion . . . . . . . . . . . . . . . . . . . . . . . 385
Treatment of the Throat, Nose and Ear in Scarlet Fever . . . . . . . . . . . . . . . 388

CORRESPONDENCE—

From Dr. Smythe . . . . . . . . . . . . . . . . . . . . . . . . . . . . . . . . . . . 392

EDITORIAL—

Rational Medicine; Assisting Nature . . . . . . . . . . . . . . . . . . . . . . . . . 393
State Health Officer's Report . . . . . . . . . . . . . . . . . . . . . . . . . . . . 396

MEDICAL NEWS AND MISCELLANY. . . . . . . . . . . . . . . . . . . . . . . . . . . 398–402
BOOK NOTICES . . . . . . . . . . . . . . . . . . . . . . . . . . . . . . . . . . . . 402–405
PUBLISHERS' NOTES . . . . . . . . . . . . . . . . . . . . . . . . . . . . . . . . . . 405–414

